# Bridging the Diagnostic Gap: A Rapid, Cost-Effective, and Equitable Hepatitis C Virus RNA Detection Method for Resource-Limited Settings

**DOI:** 10.7759/cureus.92101

**Published:** 2025-09-11

**Authors:** Shyam Prakash, Supriya Bharti, Ram Aasarey, Priyatma, Manish Saroj, Shahid Khan, Medha, Shivam Pandey

**Affiliations:** 1 Laboratory Medicine, All India Institute of Medical Sciences, New Delhi, New Delhi, IND; 2 Virology, Pandit Bhagwat Dayal Sharma Post Graduate Institute of Medical Sciences, Rohtak, IND; 3 Biostatistics, All India Institute of Medical Sciences, New Delhi, New Delhi, IND

**Keywords:** bland-altman plot, calcein, hcv rna, hnb, icc, in-house qpcr, lamp methods

## Abstract

Hepatitis C virus (HCV) remains a major global health challenge, particularly in resource-limited settings where access to molecular diagnostics is restricted. The loop-mediated isothermal amplification (LAMP) assay offers a rapid, cost-effective alternative to polymerase chain reaction (PCR) for HCV RNA detection. Unlike PCR, LAMP uses isothermal amplification (60-65°C), eliminating the need for expensive thermal cyclers and yielding results in under 60 minutes. We evaluated two LAMP detection methods - hydroxynaphthol blue (HNB, colorimetric) and calcein (fluorescent) - against an in-house TaqMan qPCR assay. Both LAMP methods demonstrated high sensitivity (99.6%) and specificity (95.6% for HNB, 99.2% for calcein), with a broad dynamic range (10-10⁶ copies/mL). Clinical validation against real-time PCR showed strong agreement, with a positive predictive value of 98.6% and a negative predictive value of 94.4%. The HNB-LAMP assay, in particular, provides a simple visual readout (blue to sky blue), requiring no specialized equipment, while calcein-LAMP offers fluorescence-based detection under UV light. Both methods showed no significant correlation (p > 0.5), confirming their reliability. LAMP’s advantages - minimal infrastructure, ambient temperature stability, and low cost (<$5 per test) - make it ideal for decentralized testing in low-resource settings. This approach could revolutionize HCV diagnosis by enabling same-day test-and-treat strategies, improving linkage to care, and supporting global HCV elimination efforts. Future steps include field validation in remote clinics, manufacturing scale-up, and integration into point-of-care platforms to maximize accessibility. By bridging the diagnostic gap, LAMP can potentially transform HCV management in underserved regions worldwide.

## Introduction

Nucleic acid amplification tests are widely used in blood banks to screen for infectious diseases (hepatitis B virus (HBV), hepatitis C virus (HCV), and HIV). However, these are not suitable for routine viral load detection. Polymerase chain reaction (PCR) and real-time PCR are the only methods that require well infrastructure, trained technical staff, and expertise in molecular diagnostics. HCV is a positive-sense, single-stranded RNA virus belonging to the Flaviviridae family. The genomic length of HCV is 9.6 kb and consists of 5' and 3' untranslated regions (UTR) at both ends and a single large open reading frame (ORF) [1,2]. The HCV strains are genetically heterogeneous and classified into six major genotypes [3]. The prevalence of the HCV genotype varies geographically in the global population. HCV genotype 3 is more prevalent in India and constitutes about 75% of infections, whereas in Asian countries, genotype 1 is more common [4]. Chronic HCV infection in the liver for a longer duration progresses to liver fibrosis, cirrhosis, and hepatocellular carcinoma (HCC) [5] if undiagnosed.

Currently, HCV antibody detection, viral RNA PCR, and quantitative PCR (qPCR) are the only available methods for detecting HCV infection, though the presence of HCV antibodies in an individual reflects a past infection. While antibody testing only reflects past exposure, confirmation of ongoing infection depends on RNA detection [6]. Thus, HCV RNA testing is still challenging in confirming ongoing HCV infection. However, PCR and qPCR nucleic acid-based detection techniques are the most reliable and available methods for detecting HCV RNA, limiting the application for onsite HCV diagnosis [7]. However, various commercially available kits for HCV RNA detection are limited to the coverage of the entire genotype in a single kit. Based on that, we have also developed an in-house method covering the genotypes of HCV RNA, particularly in the Indian scenario, genotypes 1 and 3, used fluorescent-labelled probe, and specific primers to cover the entire target sequence. Importantly, the developed LAMP assay offers a significantly faster and simpler alternative to conventional PCR and qPCR methods while still adhering to established diagnostic norms for HCV RNA detection.

Loop-mediated isothermal amplification (LAMP) is a nucleic acid-based amplification method that amplifies the target sequences in a short interval under isothermal conditions [8]. This method required specific LAMP primers (forward and backward), inner primers (forward and backward), loop primers (forward and backward), and *Bacillus stearothermophilus* (Bst) DNA polymerase for amplification of the target gene sequence in a digital water bath that furnishes a constant temperature without an expensive thermo-cycler machine [9,10]. Hydroxynaphthol blue and calcein are metal-recognized dyes that react with pyrophosphate (PPi) accumulation during the DNA amplification at isothermal conditions, depending upon the viral loads [11]. These colorimetric readouts allow rapid visual or spectrophotometric detection (515-645 nm for HNB and 490-520 nm for calcein) when compared against no-template controls. Thus, the LAMP assay represents a rapid, simple, and cost-effective alternative for HCV RNA detection, with strong potential for application in remote areas. HNB and calcein were comparatively detected by a spectrophotometer at 515-645 nm and 490-520 nm, respectively, against a no-template control in the patient samples. Thus, the LAMP assay is essential for detecting HCV infection, particularly in remote areas and urban setups with limited accessibility to HCV RNA testing. HCV infection is more prevalent, and the LAMP assay to detect HCV viral RNA covering genotypes 1 and 3 in the Indian population. Therefore, rapid, simple, and cost-effective methods for detecting HCV RNA levels are urgent to avoid the progression of cirrhosis and HCC and to help in early detection and management of HCV infection.

## Materials and methods

Recruitment of patients and selection criteria

**Figure 1:**
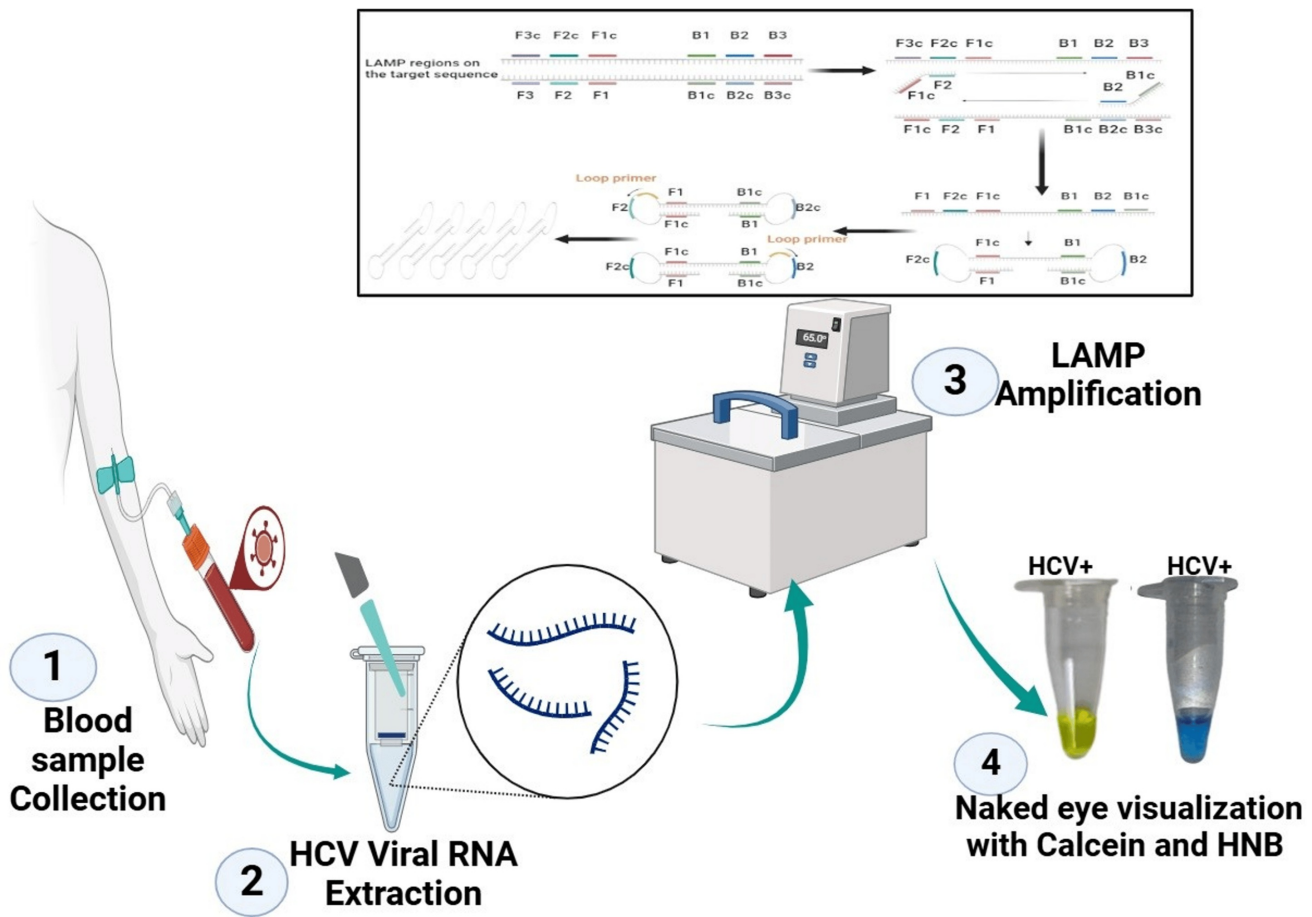
Schematic representation of HCV detection using the LAMP method. (1) Blood sample collection: A patient’s blood sample is collected for HCV testing. (2) HCV viral RNA extraction: The viral RNA is extracted from the blood sample to serve as the template for amplification. (3) LAMP amplification: The extracted RNA undergoes loop-mediated isothermal amplification (LAMP) using specific primers and a heating system to facilitate amplification. The top inset illustrates the LAMP primer binding sites and amplification mechanism. (4) Naked eye visualization with HNB dye: The results are visualized by the addition of hydroxynaphthol blue (HNB) dye and calcein, where a color change indicates a positive reaction (HCV+). This method provides a rapid, sensitive, and visual detection of HCV infection using LAMP technology.

Eight hundred thirty-eight subjects were screened from the OPD and liver clinic of the Department of Medicine, Gastroenterology and Blood Collection Centre of Laboratory Medicine at All India Institute of Medical Science (AIIMS), New Delhi, India. Venous blood collected from all recruited subjects was 5 mL, and serology was performed for anti-HCV antibody, IgM anti-HBc, Anti-HEV, and HBsAg by the commercially available enzyme-linked immunosorbent assay (ELISA) kit (Bio-Rad Monalisa; Bio-Rad, Hercules, CA). HIV 1 and HIV 2 were done by the fourth-generation ELISA kit (Genedia HIV Ag-Ab), and liver function test was done in a chemistry Autoanalyzer. As per the holistic guidelines, patients were recruited, and informed consent was obtained from all recruited subjects for the study, as per the Declaration of Helsinki. The recruited subjects were divided into two groups: (1) anti-HCV-positive and (2) anti-HCV-negative. Figure 1 provides the schematic representation of HCV detection using the LAMP method.

Sample size calculation

The sample size for this study was determined based on statistical methods appropriate for the comparison of a new diagnostic test against the standard methods to evaluate the diagnostic performance (sensitivity and specificity) of the new method (modified) for the detection of HCV RNA. The sample size was calculated keeping the given appropriate precision for the test parameters [12].

Ethical clearance was obtained from the Institute's Ethical Committee of the All India Institute of Medical Sciences, New Delhi (Study code no # IEC/NP-400/2013).

Primer design and its validation

The real-time PCR (primer and probe) assay was designed using primer designer software (Beacon Designer; Premier Biosoft, San Francisco, CA), and the primers for the LAMP assay for HCV were designed using LAMP designer software (Premier Biosoft, San Francisco, CA). The primers and probes were selected with the coverage of most of the genotypes, explicitly genotypes 1 and 3, for in-house HCV RNA quantification. All primers were synthesized from Integrated DNA Technologies (Coralville, IO). A set of forward primer (LF), loop backwards primer (LB), forward outer primer (F3), and backwards outer primer (B3) was designed keeping in view the target amplification of the gene. A complete nucleotide sequence of the primer and amplicon size is shown in Table 1.

**Table 1:** Primer sequence and target region of HCV. HCV: Hepatitis C virus

Polarity	Nucleotide Sequence (5'-3')	Target region
LAMP (PCR)		
Sense (F3) Antisense (B3)	TCACCATATTCTTGGGAACAAGA TTCCTGAACTGGAGCCACCA	2817-2839 415-396
LAMP (Inner Primer)		
Sense (FIP) Antisense (BIP)	TCATGTCCTACTGTTCAAGCC GCGAGGCGAGGGAGTTCTTCT	1850-1871 2392-2413
LAMP (Loop Primer)		
Sense (LF) Antisense (LB)	GCATGGAGACCACCGTGAAAC GGAAAGAAGTCAGAAGGCCAA	1606-1625 1974-1954
HCV (Outer PCR)		
Sense Antisense	GCATGGAGACCACCGTGAAAC GGAAAGAAGTCAGAAGGCCAA	1606-1625 1974-1954
HCV (Real-Time PCR)		
Sense Antisense Probe	TAGGAGXYTGTAGGCACAAAT AAGGAAYXAGTTTGCCATTCA FAM-CCCTCCCGGGAGXGCCATAYTG-TAMARA	1776-1796 2542-2518

The primer concentrations for both LAMP and PCR assays were optimized through a series of preliminary experiments varying primer amounts systematically to determine the concentrations yielding maximal amplification efficiency and specificity at 1.2 μL of 10 pM and 1 uL of 10 pM, respectively. This optimization step ensured reproducibility and minimized nonspecific amplification.

**Figure 2:**
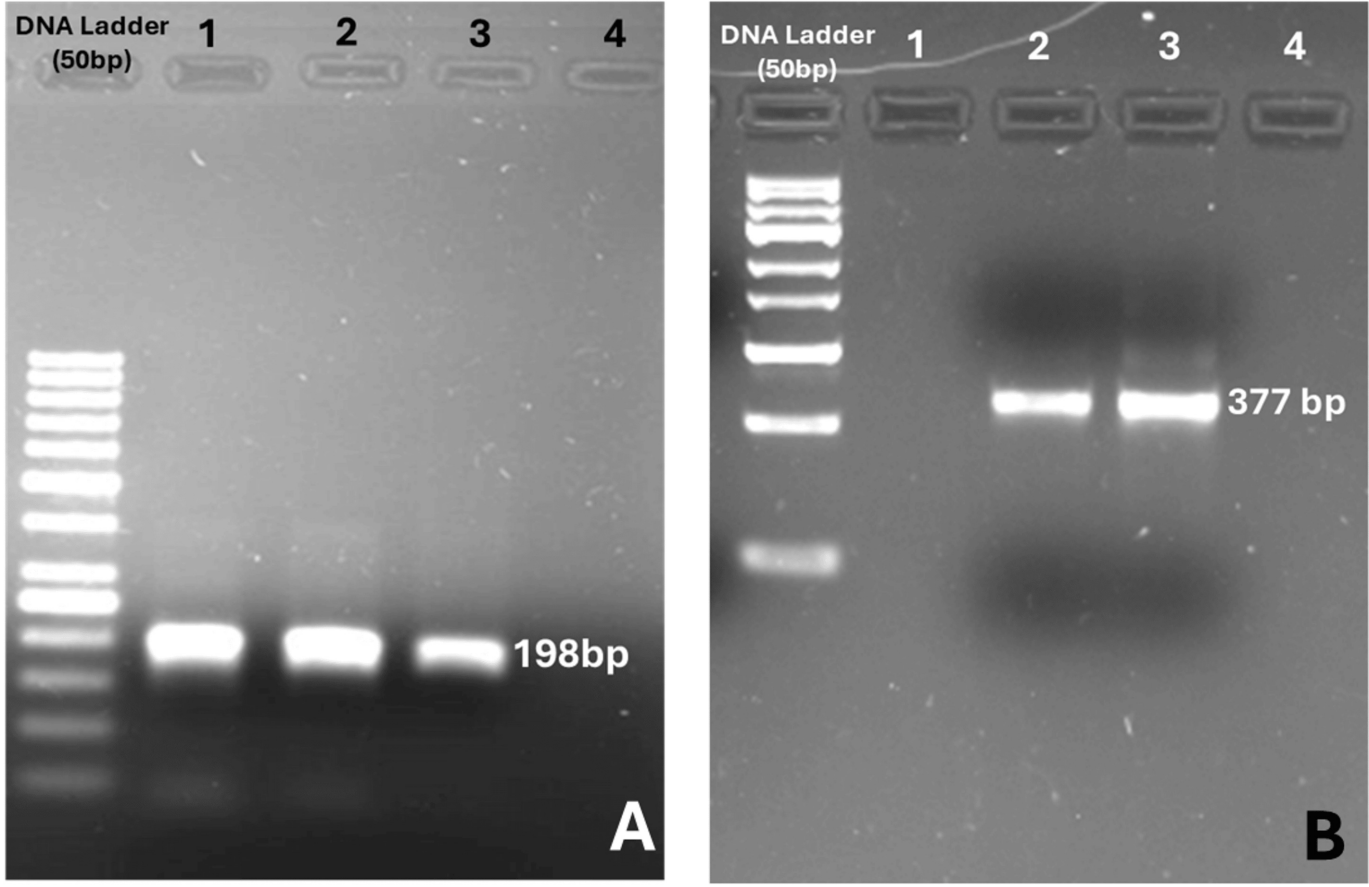
Gel electrophoresis analysis of DNA amplification using LAMP and PCR primers. (A) Gel electrophoresis results of loop-mediated isothermal amplification (LAMP) primer-based amplification. The DNA ladder (50 bp) is included for size reference. Distinct bands at 198 bp confirm successful amplification using LAMP primers (F3 and B3). (B): Polymerase chain reaction (PCR) amplification targeting HCV. The DNA ladder (100 bp) provides molecular weight markers. Clear bands at 377 bp indicate successful amplification using HCV-specific PCR primers.

The synthesized primer was run in an electrophoresis system in a 1% agarose gel containing 1 mg/dL ethidium bromide (EtBr) to check for specificity. Subsequently, the amplified product was visualized under gel documentation (Alpha Innotech, Cellbiosis, Germany) in the sample. The amplified products are shown in Figure 2.

RNA isolation and cDNA synthesis

Viral RNA was extracted from blood samples using a QIAamp viral RNA mini kit (Qiagen, GmbH, Germany). Following this, cDNA was prepared using 5 units of reverse transcriptase enzymes (Moloney murine leukemia virus (MMuLV) RT) and 5 µL RNA template and random hexamer primer, with a total of 20 µL reaction buffer at 42℃ for cDNA preparation. A total of 20 µL of the reaction mixture was prepared to contain 10x MMuLV RT buffer (New England Biolabs, Ipswich, MA), 10 mm dNTP mix, and 20 U/µL RNase inhibitor, and volume makeup was done by using nuclease-free water. Incubation of the reaction for the conversion to cDNA was done at 42°C for 30 minutes and 65°C for five minutes to complete the reaction process for the cDNA complete synthesis.

Quantification of HCV RNA by real-time PCR

The designed primer and probe by Beacon Designer software and synthesized from Integrated DNA Technologies (IDT), Canada, on a 100 nm scale, were used to amplify HCV RNA. The probes were labelled (FAM & TAMRA) using TaqMan chemistry for amplification in real-time PCR. The reaction mixture was performed using master mix (2x); primers (forward and reverse); FAM- and TAMRA-labelled probes with thermal cycling conditions at 95°C for 10 min (one cycle), 95°C for 15 seconds, and 60°C for 60 seconds (45 cycles); and qPCR (Agilent Technologies, Santa Clara, CA). The WHO and AcroMetrix HCV standards were used to assess the complete amplification of HCV RNA and to calculate the amplification expression of target genes. The sensitivity and efficiency were calculated as R² 1 or < 0.95, and the efficiency range was 80-100% by the software analysis.

LAMP reaction and product detection

A total of 20 µL of reaction mixture was prepared, containing 1.6 µM each of forward loop primer (FLP) and backward loop primer (BLP), 0.2 µM each of F3 and B3, 0.4 µM of FLP and BLP, 10.5 µL of master mix (2x, Affymetrix), 16 mM MgSO_4_ and 0.01% Tween 20, 10 mM deoxynucleotide triphosphate (dNTP mix), 1M Betaine (Sigma-Aldrich, Burlington, MA), 8U Hot-start Bst DNA (New England Biolabs), 5U MMuLV RT, 5 uL of template (cDNA), and 150 µM hydroxynaphthol blue (HNB) and calcein (Dojindo, Japan). The reaction mixture was incubated at 60°C for one hour in the heating device (IKA, digital dry block; BOECO Germany, Germany). After that, 5 µL of the LAMP-amplified product was run in an electrophoresis system in a 1% agarose gel containing 1 mg/dL ethidium bromide (EtBr). Subsequently, the amplified product was visualized under a gel documentation system (Alpha Innotech, Cellbiosis, Germany). The positive LAMP product appeared as a typical ladder pattern with multiple bands of different sizes on a 1% agarose gel, as shown in Figure 2.

The LAMP product was also seen by the naked eye, and this reaction was on the color change in the reaction mixture by HNB induction, depending on the concentration of the viral load in the observed reaction mixture. It binds to Mg^2+^ and forms a purple color. During the synthesis process, the concentration of Mg^2+^ was decreased due to binding with its pyrophosphate accumulation in the reaction mixture. The amplified LAMP product, if positive, turned sky blue, and if negative, the amplified product remained purple. During amplification with calcein, the precombined Mn^2+ ^ions are converted into pyrophosphate ions (PPi−) due to DNA synthesis. As a result, the assay changes from bright orange to light green when exposed to natural light, regaining the quenched green fluorescence of calcein [13].

Statistical analysis

A confusion matrix was employed to compare the performance of LAMP and real-time PCR, enabling the calculation of sensitivity, specificity, accuracy, and likelihood ratios (LR). Agreement between the two diagnostic methods was further assessed using the Bland-Altman analysis and ICC, which assumes that both methods yield comparable results for each measurement instance [14,15]. A scatter plot was generated to visualize the quantitative relationship between the variables measured from the same sample set [16,17]. The Pearson correlation coefficient (r) was also calculated to evaluate the strength of the linear association between the two methods. All statistical analyses were conducted using Microsoft Excel (Microsoft® Corp., Redmond, WA), Statistical Product and Service Solutions (SPSS, version 25.0; IBM SPSS Statistics for Windows, Armonk, NY), and R Studio (RStudio Team, Boston, MA).

## Results

Patient's selection and serology

A total of 838 subjects were screened for HCV RNA. Eighty subjects were found to be positive for HCV RNA. The remaining 340 subjects were positive for hepatitis B surface antigen (HBsAg), 12 subjects for anti-hepatitis E (HEV), 316 subjects for IgM anti-HBc, and eight subjects were positive for HIV 1 and HIV 2 infection. Eighty subjects were found to be negative for all serology tests; that is, they were neither positive for HBV, HIV, and HCV infection. Their serum glutamate transaminase (SGPT) and serum glutamate oxalate transaminase (SGOT) levels were deranged (75-400 U/mL), and bilirubin levels were found from 1.8 mg/dL to 2.6 mg/dL. Based on serology, subjects (n=80) were further categorized for the LAMP assay for HCV RNA amplification in the samples.

HCV RNA by real-time PCR (qPCR)

Viral RNA was extracted using the Qiagen viral RNA extraction kit using the manufacturer's protocol. The purity and yield of extracted RNA were measured using a NanoDrop (Genetix Biotech Asia Ltd., India). The obtained RNA was used for the quantification of HCV RNA by the following methods.

*qPCR Using In Vitro Diagnostic *(*IVD) Kits*

Quantifying HCV viral RNA was done using LiferiverTM Technologies (Bio-Tech Co. Ltd., Shanghai, China) as per the manufacturer's protocol (IVD-approved kit). RNA template was added to the master mix containing 13 µL super mix, 1 µL enzyme mix, and 1 µL internal control. Then, the reaction was initiated in the thermal cycler (Agilent Technologies, Inc., Santa Clara, CA) at 45°C for 10 minutes, denaturation at 95°C for 15 minutes, and 40 cycles of 95°C for five seconds and 60°C for 30 seconds.

Development of an In-House Real-Time PCR Method

The total reaction was performed by using 2x master mix (USB Affymetrix), FAM- and TAMRA-labelled probe (10 pM), primers (forward and reverse) synthesized from IDT Canada, keeping in view of the target region (5’UTR) for complete reaction of HCV RNA amplification in qPCR (Agilent Technologies). The WHO and AcroMetrix® HCV standards were used to generate standard curves at various ranges of dilutions (101-105 IU/mL). The thermal reaction condition was kept at 95°C for 10 min (one cycle), 95°C for 15 seconds, and 60°C for 60 seconds (45 cycles) to amplify HCV RNA in blood samples.

**Figure 3:**
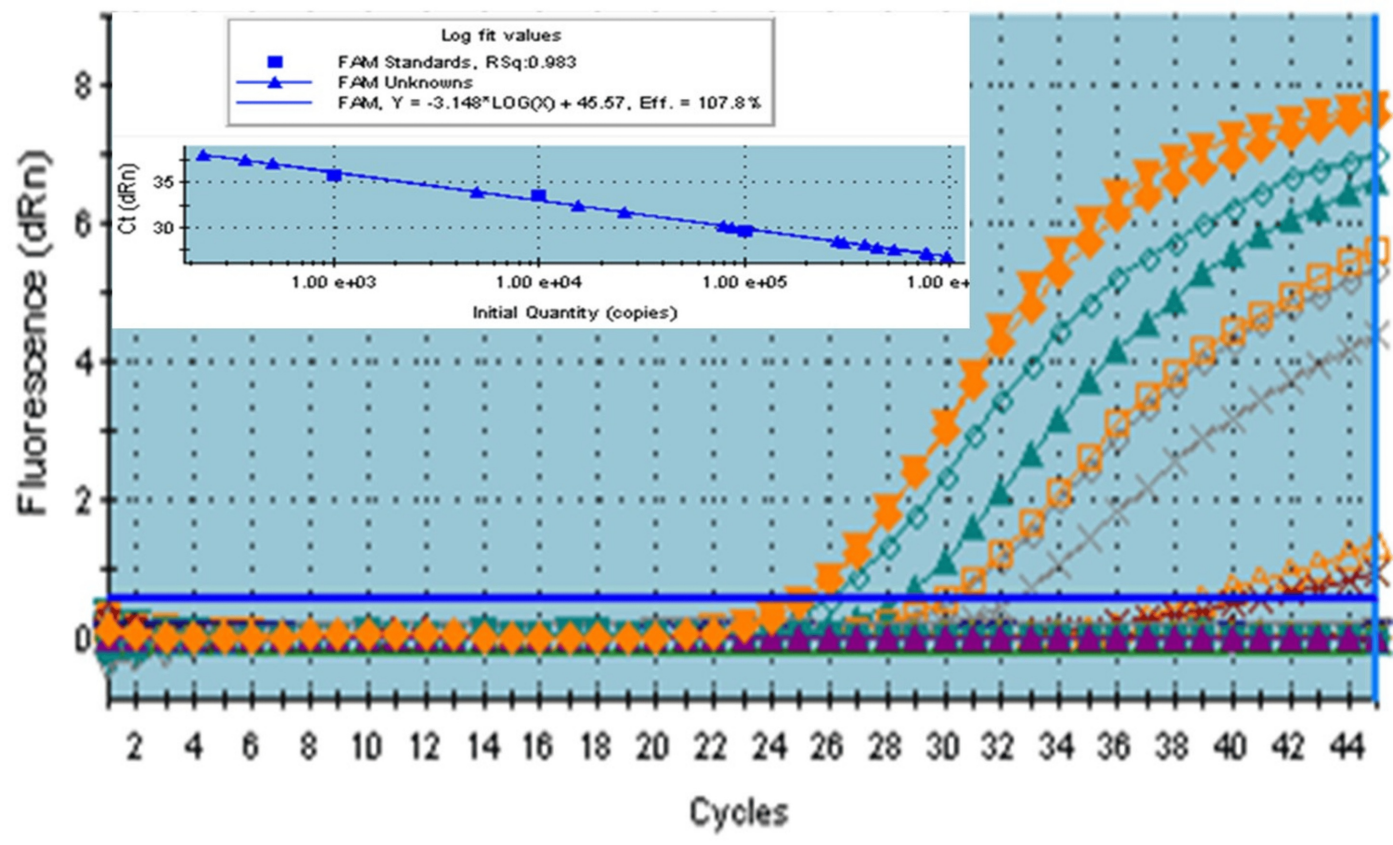
The amplification plot represents fluorescence intensity (dRn) as a function of the number of polymerase chain reaction (PCR) cycles. Each color in the plot corresponds to a different sample or condition, with curves representing the amplification process over time.

The same RNA samples were processed for LAMP reaction assay to quantify viral RNA and quantified by ELISA reader at 645 nm using HNB and 515 nm using calcein. The amplification plot of HCV viral RNA is shown in Figure 3.

Among 80 HCV-positive samples, all were identified as positive using the in-house method and IVD kits. One sample tested positive with the in-house method for the HCV-negative samples, while the remaining 79 samples were tested negative by both methods. The sensitivity and specificity of the in-house method were calculated up to 99% (95% CI: 0.93-1.00), using a confusion matrix.

LAMP assay development

To test the amplification of HCV RNA in the LAMP assay, which does not require DNA denaturation steps to amplify the target gene, we used two metal indicator dyes for the LAMP reaction to assess whether the amplification is better or higher for quantification than PCR.

HNB Dye

**Figure 4:**
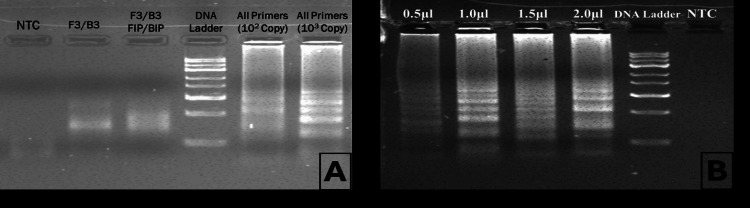
Gel electrophoresis analysis of DNA amplification under different conditions. Evaluation of primer efficiency and amplification success. Lanes include no template control (NTC), individual primer sets (F3/B3, FLP/BLP), a DNA ladder for size reference, and amplification using all primers with 10² and 10³ DNA copies, showing distinct bands indicating successful amplification. The absence of bands in the NTC confirms no contamination. (B) Optimization of primer concentration. Lanes represent reactions with increasing primer volumes (0.5 µL, 1.0 µL, 1.5 µL, and 2.0 µL). The intensity of the amplified bands increases with primer volume, suggesting an optimal range for efficient amplification. A DNA ladder is included for size comparison, and the NTC shows no amplification, ensuring reaction specificity. These results validate primer performance, amplification efficiency, and optimal conditions for DNA detection.

Various sets of reactions were performed at different time intervals and primer concentrations to optimize the amplification of target sequences. Thus, the LAMP reaction was optimized at 60°C for 60 minutes of incubation with variable concentrations of loop, inner and outer primers, and 150 µM of HNB in the dry heating bath for rapid reaction and visual detection of HCV RNA, as shown in Figure 4.

**Figure 5:**
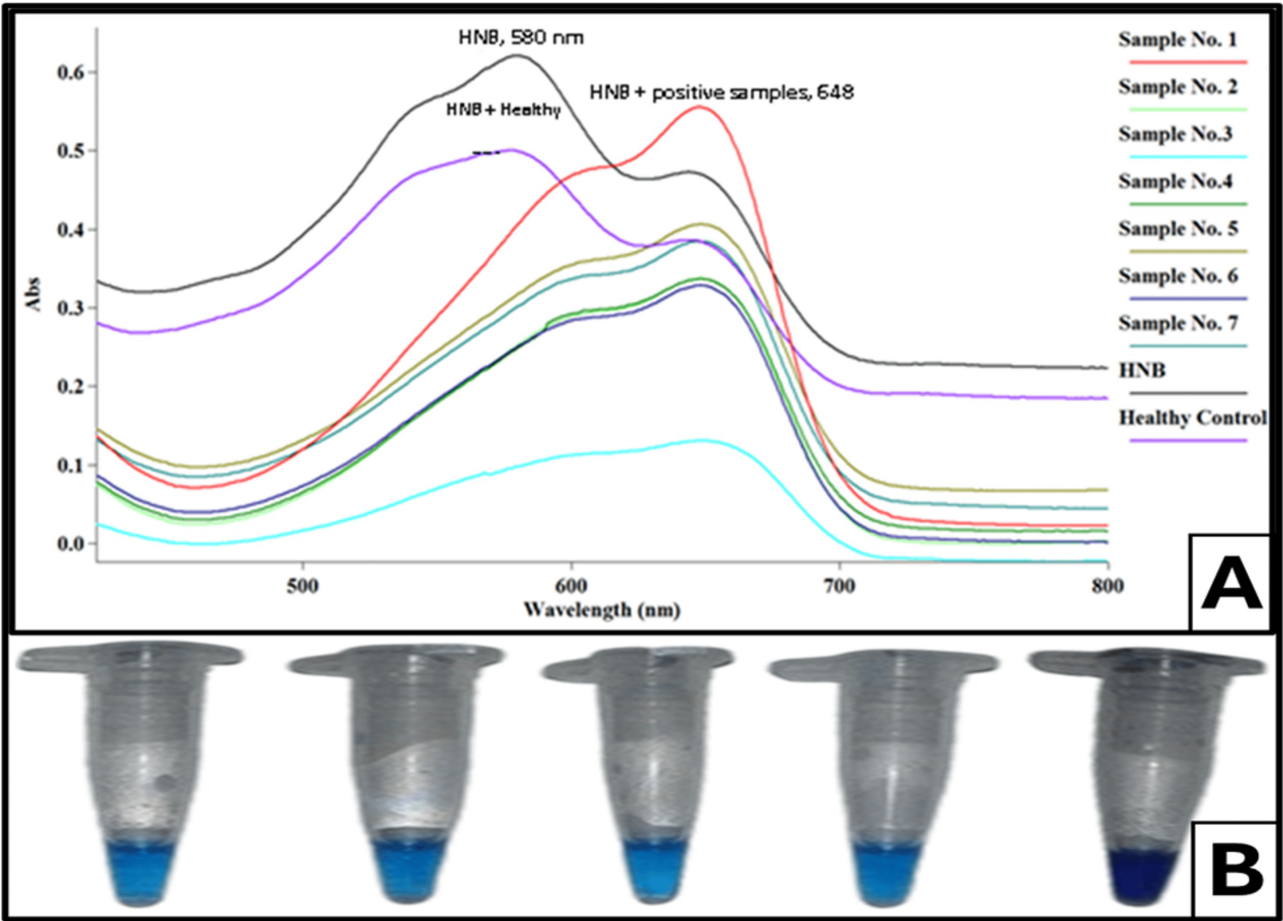
Spectrophotometric analysis and visual detection of hepatitis C virus (HCV)-positive and healthy samples using hydroxynaphthol blue (HNB) dye. (A) UV-vis absorption spectra of LAMP-amplified HCV samples with HNB dye, showing distinct absorbance peaks at 580 nm for the healthy control and 648 nm for HCV-positive samples. The color shift in absorbance spectra differentiates positive and negative samples. (B) Visual detection of HCV amplification in LAMP reaction tubes. A distinct color change from violet (negative) to sky blue (positive) confirms the presence of HCV RNA in the test samples. This simple, naked-eye detection method facilitates rapid and reliable identification of HCV-positive cases.

Similarly, along with the reaction mixture, different concentrations of HNB were also tested in a single tube for the visualization of LAMP products. The sky blue color was indicated in samples that were positive for HCV RNA, and the purple color showed a negative for HCV RNA in samples in HNB, as shown in Figure 5.

Calcein Dye

**Figure 6:**
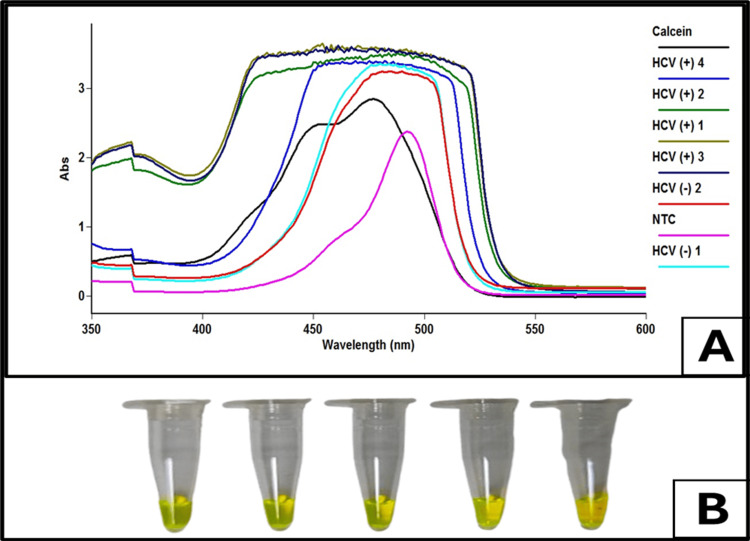
Spectrophotometric analysis and visual detection of hepatitis C virus (HCV)-positive and negative samples using calcein dye. (A) UV-vis absorption spectra of LAMP-amplified HCV samples in the presence of calcein dye, showing distinct absorbance patterns. Positive samples (HCV+) exhibit higher absorbance intensities, while negative samples (HCV−) and the no-template control (NTC) display lower absorbance values. (B) Visual detection of LAMP-amplified HCV samples in reaction tubes. A color change to bright yellow indicates successful amplification in HCV-positive samples, whereas negative samples and NTC remain unchanged. This method enables rapid, naked-eye detection of HCV infection.

Various concentrations of calcein dye were used and accordingly fixed at a concentration for the reaction mixture. Similarly, the calcein assay changes from bright orange to light green when exposed to natural light, as shown in Figure 6.

Of the 80 HCV-positive samples tested by LAMP methods with two dyes (i.e., HNB and calcein), 79 were found positive by both the HNB and calcein dyes, while one sample was found negative by calcein dye. Among the 80 HCV-negative samples, four samples were found positive with the calcein dye, whereas the remaining 76 samples were found negative by both dyes. Based on the confusion matrix analysis, the sensitivity of calcein dye was 95% (95% CI: 0.88-0.99), and the specificity was 99% (95% CI: 0.93-1.00).

Clinical validation of LAMP assay with qPCR

**Figure 7:**
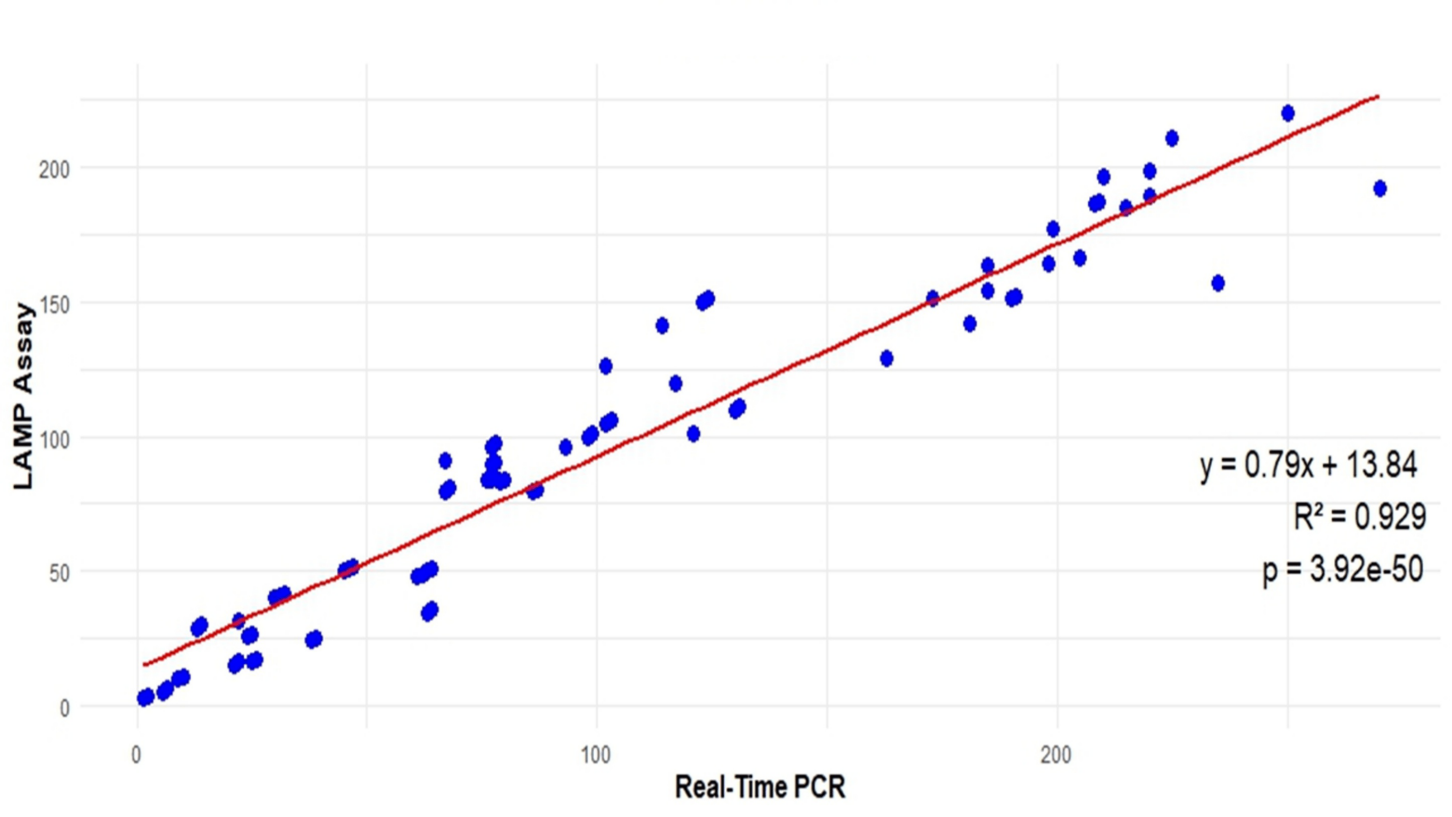
Scatter plot illustrates the correlation between the loop-mediated isothermal amplification (LAMP) assay and real-time polymerase chain reaction (PCR) for quantifying hepatitis C virus (HCV) samples. Each data point represents an individual sample analyzed by both methods. The coefficient of determination 𝑅^2^=0.929 indicates a strong positive correlation, demonstrating the reliability of the LAMP assay as a quantitative tool for HCV detection.

All recruited subjects were initially tested for HCV RNA by qPCR and later by LAMP assay. The sensitivity and specificity of the LAMP method concerning the in-house developed qPCR method were analyzed using a confusion matrix. Seventy-eight samples were positive for both methods, while two were negative by the LAMP method. Similarly, for HCV-negative samples, 79 samples were found negative for both methods, while one sample was found positive for the LAMP method. The specificity of the LAMP assay for HCV RNA detection was found to be 99% (95% CI: 0.93-1.00), and sensitivity was up to 98% (95% CI: 0.91-1.00). The correlation coefficient (r) for the LAMP assay and qPCR is ~93%, which indicates a very strong relationship between these two methods, as shown in Figure 7.

Method agreement between the LAMP assay and qPCR

To assess the agreement between the LAMP assay and qPCR, we employed both the intraclass correlation coefficient (ICC) and Bland-Altman analysis.

Intraclass Correlation Coefficient (ICC)

**Figure 8:**
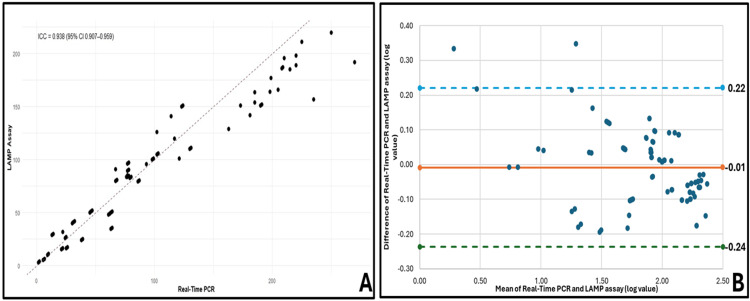
The agreement between LAMP assay and real-time PCR. Agreement was assessed by both intraclass correlation and Bland-Altman methods on log-transformed viral load measurements. (A) Scatter plot with ICC annotation. Each black dot represents one sample’s loop-mediated isothermal amplification (LAMP) assay result plotted against its corresponding real-time PCR value. The red dashed line is the line of identity (y = x). The one-way random-effects intraclass correlation coefficient is ICC (1, 1) = 0.938 (95% CI: 0.907-0.959), indicating excellent absolute agreement between the two methods. (B) Bland-Altman plot. The difference (PCR - LAMP) is plotted against the mean of the two methods for each sample. The central red line shows the mean bias (-0.01 log₁₀ units), and the upper (purple) and lower (green) dashed lines indicate the 95% limits of agreement (-0.24 to 0.22 log₁₀ units). Most points lie within these bounds, demonstrating minimal systematic bias and tight concordance across the full measurement range.

The ICC (1,1) was calculated to measure the absolute agreement between the two methods, using a one-way random effects model based on a single measurement from each method. The ICC value 0.938 (95% CI: 0.907-0.959) reflects excellent agreement between the LAMP assay and qPCR across the tested viral load range. This high level of consistency indicates that both methods yield highly concordant results. The corresponding scatter plot (Figure 8A) shows each sample’s LAMP value against the PCR value, with a red dashed identity line (y = x) representing perfect agreement. Although most data points align closely with the trend line, minor deviations occur, particularly at the highest and lowest viral loads, indicating slight variability

Bland-Altman Analysis

A Bland-Altman plot was created to complement the ICC results to visualize the differences between the two methods across all measurements. In this analysis, the difference between the qPCR and LAMP assay (log₁₀-transformed) was plotted against the mean of the two methods. The mean bias (difference) was calculated as -0.01 log units, indicating negligible systematic bias between the methods. The 95% limits of agreement were found to be -0.24 to 0.22 log units, meaning that most data points lie within these bounds, confirming that the methods are interchangeable with only a minor, clinically insignificant difference, as shown in Figure 8B. This further reinforces the conclusion from the ICC analysis that the two methods exhibit robust agreement.

Cross reactivity

To test the specificity of the LAMP assay for the detection of HCV RNA, HBV-positive, and HIV-positive samples were used as templates for the cross-reactivity of the assay, if any. HCV-positive samples were amplified, while no amplifications were observed in other viral (HBV and HIV) infected samples. A typical ladder pattern was found only in HCV-positive samples. At the same time, no band was detected in either non-specific viral template (HBV and HIV) or negative samples. The amplified product was visualized with the naked eye and checked in the gel electrophoresis system for the sensitivity and reproducibility of the LAMP assay for HCV RNA detection. The pre-addition of HNB dye in the reaction mixture minimized the contamination risk and, subsequently, depending upon the viral load, enables direct visual detection of amplification without requiring gel electrophoresis or UV equipment, improving assay suitability for resource-limited and decentralized settings.

## Discussion

HCV is a significant public health problem and a leading cause of chronic liver disease. The great majority of HCV-infected individuals are asymptomatic or unaware of their infection. Therefore, early detection of HCV infection is crucial for initiating antiviral treatment and controlling the disease progression. Globally, an estimated 58 million individuals are chronically infected with HCV, and the WHO has set a new goal of reducing approximately 90% of new HCV infections and 60% of mortality by 2030. Rapid detection and accurate identification of HCV in patients at all infection stages are crucial for initiating proper antiviral treatment. Clinical diagnostics are based on antibody detection assays [18]. The serology method has several disadvantages due to the need for test repeatability. Nucleic acid-based detection is the available method for detecting and quantifying HCV viral RNA. Unfortunately, qPCR-based methods require high-precision instrumentation and elaborate methods for detecting viral infection [19]. Thus, qPCR methods have been avoided, especially in peripheral healthcare settings, private clinics, and remote areas [20]. Real-time PCR is a better quantifying tool for DNA or RNA concentrations; hence, we developed an in-house method for HCV RNA quantification to cover the entire range of genotypes, specifically genotype 1 and genotype 3, which are widely distributed in the Indian scenario. Our results were comparable to those of IVD kits for HCV RNA viral detection, with the sensitivity and specificity of 99% (95%CI: 0.93-1). However, the precise minimum copy numbers detectable using in-house methods were 50 copies/mL, compared to the IVD-approved real-time kits, which are more than 300 copies/mL in the patient sample. The key difference between a commercially available kit and an in-house qPCR is worth discussing, as one is more reproducible and accurate for detecting HCV RNA load. Another key difference is that the in-house assay covers genotypes 1 and 3, which is more appropriate for early diagnosis of the Indian population. In response to these issues, we developed the HCV LAMP assay to achieve the detection of HCV RNA in clinical samples. Compared to qPCR, the LAMP approach is significantly more straightforward, and its detection ability is more sensitive [8,20]. The significant advantages of the LAMP are its high specificity (since it uses six primers recognizing eight distinct regions on the target sequence), high sensitivity, and rapidity under isothermal conditions (two hours, including the extraction step, compared to three to four hours for the qPCR assay). The reverse transcription and DNA amplification have been performed in a single-step reaction in the tube for the RT-LAMP assay. Notably, the LAMP assay demonstrated comparable sensitivity (79.6%) and specificity (up to 83.7%) relative to RT-PCR, with the added benefits of simplicity, cost-effectiveness, and faster turnaround time (50-70 minutes post-extraction), facilitating more practical clinical and surveillance applications. In addition, an attractive property of the RT-LAMP assay is that the results of RT-LAMP products can be observed immediately with the naked eye by active incorporation of the HNB dye.

HNB was pre-added in the RT-LAMP reaction in a closed tube to avoid carry-over contamination in the post-amplification process. HNB allows visual detection with the naked eye without equipment or a UV lamp, and we confirmed that the results of visual detection were equal to those of gel electrophoresis. Notably, the pre-addition of HNB in the RT-LAMP reaction did not inhibit amplification efficiency [21]. Therefore, without other intercalating dyes and a turbidimeter, HNB would be ideal for use in resource-limited settings, as it is low cost, and the results can be judged easily by the color change. Similarly, calcein is another Mn^+2^-associated quenching fluorophore that provides yellow under visible light at the reaction point from orange due to an increased concentration of PPi, depending upon the viral load in the sample. No thermal cycler instrument is required in the LAMP assay because no heat denaturation step is used with the template DNA.

To determine the test's detection limit, HCV RNA serial dilutions were tested by RT LAMP. However, the serial dilution of HCV RNA in this study differed from that in a previous study [22]. Regarding the measurement unit, the comparison is more complicated. In the study by Nyan et al. [22], the HCV RNA serial dilution ranged from 10 to 0.1 IU per reaction, with a detection limit of 100 IU per reaction (100% detection rate). In contrast, the LAMP assay showed good inter- and intra-assay reproducibility over a wide dynamic range (50 x 10^1^ to 1 x 10^6 ^IU/mL).

Our results demonstrated that LAMP has a high sensitivity (~79.6%, equal to that for the RT PCR method and specificity was calculated up to 83.7%. The results obtained from serial 10-fold dilutions of RNA from positive clinical samples showed that both systems could detect low viral load as ten copies/mL. After the LAMP reaction, we quantify the higher accumulation of DNA concentration by NanoDrop. The sensitivity of detecting HCV RNA by the LAMP reaction was equivalent to the PCR test, and it is considered robust because it is a more straightforward, cost-effective, and time-effective procedure. The ability to obtain accurate results within 50-70 minutes after extraction of the viral RNA will contribute to laboratory-based surveillance studies. Although the sensitivity of detecting HCV by the LAMP reaction was equivalent to the PCR test, it is considered superior because it is a simpler, cost-effective, and time-effective procedure. The ability to obtain accurate results within 50-70 minutes after extraction of the virus will contribute to laboratory-based surveillance studies. The LAMP method offers significant advantages for HCV RNA quantification, making it particularly valuable for resource-limited settings. Unlike PCR, LAMP does not require complex equipment, as it amplifies DNA at a constant temperature (60-65°C) using simple heating blocks or water baths. Detection is straightforward, using colorimetric dyes (e.g., HNB) or fluorescent indicators (e.g., calcein), eliminating the need for expensive qPCR machines. LAMP demonstrates high sensitivity (>99%) and specificity (~95-99%), comparable to PCR, while detecting viral loads as low as 10 copies/mL, which is crucial for early diagnosis and monitoring. Results are available in under an hour, enabling same-day treatment decisions - a significant advantage over PCR, which takes several hours. The method is also cost-effective, using cheaper reagents and avoiding the need for advanced laboratory infrastructure. Another key benefit is its ease of use, requiring minimal technical training, making it suitable for community health workers and point-of-care (POC) settings. Future advancements could integrate smartphone-based detection, lyophilized (room temperature stable) reagents, and multiplexed testing (HCV + HIV + HBV) to enhance accessibility further.

LAMP is highly scalable for mass screening and is ideal for high-burden regions with limited healthcare resources. Its portability and adaptability to dried blood spots (DBS) or microfluidic devices could revolutionize HCV testing in remote areas. Future innovations, such as clustered regularly interspaced short palindromic repeats (CRISPR)-enhanced LAMP or AI-assisted interpretation, could also improve accuracy and usability. Future advancements could integrate smartphone-based detection, lyophilized (room temperature stable) reagents, and multiplexed testing (HCV + HIV + HBV) to enhance accessibility further. With its speed, affordability, and simplicity, LAMP has the potential to become a game-changing diagnostic tool for global HCV elimination efforts, particularly in low- and middle-income countries (LMICs). Further development of POC-compatible formats and regulatory approvals (WHO/FDA) will be essential to maximize its impact and support the WHO’s 2030 HCV elimination targets.

Limitations

Despite a large sample size and validation against qPCR, the assay has yet to be validated in real-world resource-limited settings, where factors such as reagent stability and regulatory approvals need evaluation. Operator variability and training may affect assay consistency in decentralized settings. Cross-reactivity testing was limited to HBV and HIV, so potential interference from other pathogens requires further study. Additionally, scalability, reagent shelf-life, and testing on different sample types, such as DBS, need more investigation to ensure broad clinical application.

## Conclusions

This study validates a LAMP assay for HCV RNA quantification that demonstrates better analytical sensitivity (50 copies/mL) compared to commercial IVD kits (>300 copies/mL) while maintaining high specificity (~99%). The assay's endpoint detection, facilitated by HNB or calcein dyes for visual assessment, performs equivalently to a reference in-house qPCR method.

The key advantages of this LAMP assay are its operational simplicity, rapid reaction time, and minimal equipment requirements. By eliminating the need for electrophoresis, real-time PCR instrumentation, and highly specialized personnel, it presents a practical and cost-effective diagnostic tool ideally suited for resource-limited settings and high-throughput clinical laboratories.
